# Do we understand the real-world consequences of a future DSM?

**DOI:** 10.1371/journal.pmen.0000569

**Published:** 2026-02-27

**Authors:** Lars Veldmeijer, Jim van Os

**Affiliations:** 1 Care and Innovation in Psychiatry, NHL Stenden University of Applied Sciences, Leeuwarden, The Netherlands; 2 Division Neuroscience, Utrecht University Medical Center, Utrecht, The Netherlands; PLOS: Public Library of Science, UNITED KINGDOM OF GREAT BRITAIN AND NORTHERN IRELAND

## Background

The strategy for the future *Diagnostic and Statistical Manual of Mental Disorders* (DSM) has recently been announced [1]. Proposed changes include a shift toward dimensional approaches; increased attention to possible causal factors ranging from cultural and environmental to biological; greater emphasis on patient-reported quality of life; an expanded educational and advocacy role; and a renaming of the manual. In this opinion article, we respond by focusing on the potential real-world consequences of a future DSM. We do so by demonstrating how DSM-thinking can operate as a potential identity intervention in practice.

We present two perspectives. One is grounded in lived experience of mental distress and of receiving care within a DSM-based mental health care system in a high-income country (LV). The other is written from the perspective of a psychiatrist working within that same system (JvO), drawing on clinical experience and reflecting on the testimonial of the person with lived experience. The final section was written in co-creation. By allowing these perspectives to meet, we aim to draw attention to consequences of DSM-thinking that remain difficult to see when discussions about its future are conducted primarily at a conceptual or technical level [2]. However, it should be noted that these perspectives reflect the authors’ views and experiences and are not meant to represent the full diversity of lived or clinical experiences.

## Lived experience perspective – author 1 (LV)

“Yes, you have an autism spectrum disorder”

During the COVID-19 pandemic, I experienced persistent stress and anxiety due to prolonged isolation and the loss of everyday social contact. This became so intense that it prompted me to seek mental health care for the first time in my life. An initial online assessment with a clinician felt reassuring. However, extensive symptom questionnaires that framed my experiences in terms of severity proved to be very demanding and intensified my anxiety a lot. Two weeks after the initial assessment the clinician introduced a possible autism spectrum disorder as the cause of my complaints. At the time, this felt as a relief, and I began to use the classification as an explanatory framework for my struggles. While awaiting the formal diagnostic outcome, I increasingly reinterpreted my past with this narrative, as I believed it helped me to understand what was happening to me and why.

“Yes,” was the verdict after the lengthy diagnostic procedure, “you have an autism spectrum disorder.” I was almost cheering. I felt excited that the clinicians’ hypothesis had finally been confirmed. “How does that make you feel?”, the mental health professional asked. I replied that I had already suspected it and that it felt as though a new life was about to begin, one in which I would no longer get stuck because I could take my disorder into account, in which I finally understood why I had certain difficulties and challenges. “Is it treatable?” I asked. But no. It was a ‘neurobiological condition’ that I would have to learn to live with.

For a while, roughly half a year, this narrative proved helpful, and I made several changes in my life to align with it. However, these changes had a major impact on my quality of life. I preferred not to teach classes anymore, as they involved too many stimuli. Receiving spontaneous visitors at home? Only if scheduled, otherwise it would be too unpredictable for me, and I would grow restless. A pleasant day out in a busy city? Only if I could return to the hotel room several times a day to recharge. Slowly but steadily came the realization that I had developed behaviours that I had not exhibited before the clinician introduced the classification as a possible explanation for my complaints.

During this half-year period, I had regular conversations with my professional carer and discussed these behaviours, as they were causing me to have increasing levels of stress and anxiety. Whereas I had initially sought help because I found myself isolated at home, deprived of the human contact that had previously allowed me to live a meaningful life, I now began to organise that same life in so as to minimise human contact as much as possible, because social activity could lead to stimuli, and stimuli could exacerbate my stress and anxiety.

Over the course of this half year, my need for help continued to grow. I became more stuck than I had been before entering the mental health care system, and the care itself became more intensive. Why was I drifting further and further away from who I was? I had not only come to view my past through the lens of an autism spectrum disorder classification, but I had also begun to shape my entire future around it. Given its severe impact on my well-being, I ultimately had the classification removed from the system.

## Psychiatrist perspective – author 2 (JvO)

“Psychiatry must, and can, do better”

The clinical encounter in psychiatry is marked by an asymmetry that is easy to underestimate. The person seeking help is often searching for coherence, for a way to make sense of experiences that feel overwhelming and uncontrollable. The clinician, embedded in an institutional system, is positioned as an expert who can offer that coherence. In that context, diagnostic language carries a weight that far exceeds its formal epistemic and scientific status. I will try to substantiate this by using my own clinical experience as a psychiatrist.

What first stands out to me in this testimonial is how the classification actively reshaped everyday life and that behaviours that were peripheral became foregrounded. Sensitivity turned into limitation; avoidance became precaution; social withdrawal was reframed as necessary self-protection; life, in turn, was organized entirely in line with the description of the classification. When the diagnostic story took hold, it became a central reference point, a framework through which everyday life and a wide range of experiences was understood, strongly reinforced by dominant cultural and scientific discourses surrounding neurodevelopmental conditions and mental suffering.

From my experience, such developments are difficult to see as problematic from within the clinical encounter. They often appear consistent with psychoeducation and with prevailing ideas about accommodation and self-management. Yet, as this testimonial makes clear, the cumulative effect can be a narrowing of agency and a growing distance from one’s earlier ways of being. The person seeking help gradually becomes someone who needs more help because the explanatory framework – the diagnostic story psychiatry has introduced – has restructured the field of what feels possible in terms of personal recovery and growth. Alarmingly, I have encountered numerous such testimonies over the years.

In everyday practice, most clinicians attend to safety, context, history, motivation and talents, practical constraints, and focus on relational processes as central mechanisms of change to help someone in distress to regain a sense of direction in life. Diagnosis operates largely in the background, as an administrative requirement and as a shared language. At the same time, as a meaning-making tool, it exerts a profound influence on a person's life narrative, identity, expectation, and imagined futures, particularly when framed in reductionistic terms.

While we as clinicians are trained to emphasise that diagnoses are descriptive and routinely explain that they do not capture the person as a whole and lack explanatory depth, diagnostic procedures communicate a different message to people in need. Standardised questionnaires, comparisons to population averages, lengthy diagnostic trajectories, and formal verdicts like “you have ‘*it*’”, delivered from an expert perspective, all contribute to a sense of objectivity and finality – it gives the diagnostic story, particularly the introduced classification, considerable weight. Within that environment, I think it is entirely understandable for people with persistent distress to begin reinterpreting their past and reorganising their future around such a powerful narrative. It is also deeply problematic. Psychiatry must, and can, do better.

## Future of DSM: Co-producing the experiences it seeks to describe?

As illustrated by the two distinct perspectives, the DSM describes and influences how we understand mental suffering. The current reform agenda focuses on improving reliability, integrating potential biomarkers, and expanding lived experience participation, but remains silent on this exact mechanism of diagnostic classifications as potential identity interventions that actively co-produce the experiences they are designed to describe [1]. While formally modest in its claims, the DSM can operate in practice as a system that powerfully shapes self-understanding [2].

Across high-income countries, DSM-based systems now classify roughly 25% of the population as having a mental disorder each year, with notably high rises among young people [3]. For people who are suffering, DSM-classifications can feel both alleviating and explanatory, even when they are not intended to be. At the same time, when such descriptions are internalized as explanations for one’s suffering, they can profoundly shape who a person becomes, since people in distress often strongly desire to understand what is happening and to relieve their suffering [4]. This mechanism can affect people’s self-understanding because it assigns meaning and coherence to complex context-dependent human experiences at a moment of heightened receptivity and subsequently becomes embedded in the context of a person’s entire life. In this way, living with the understanding that a DSM-classification accounts for one’s suffering, and interacting with others and one’s environment through its lens, can give rise to new kinds of experiences that may ultimately feed back into diagnostic descriptions themselves [4].

The developers of a future DSM need to work in partnership with people with lived experience, as they possess indispensable knowledge about the phenomenology of suffering and the real-world impacts of how diagnostic systems function in practice and can affect people’s sense of self [2,4,5]. Meaningful engaging with experiential knowledge in redesigning the DSM and the story it tells about experiences of mental distress is a scientific necessity for a field that remains limited in its understanding of mental suffering [6,7].
